# A soft voting ensemble classifier for early prediction and diagnosis of occurrences of major adverse cardiovascular events for STEMI and NSTEMI during 2-year follow-up in patients with acute coronary syndrome

**DOI:** 10.1371/journal.pone.0249338

**Published:** 2021-06-11

**Authors:** Syed Waseem Abbas Sherazi, Jang-Whan Bae, Jong Yun Lee

**Affiliations:** 1 Department of Computer Science, Chungbuk National University, Cheongju, Chungbuk, South Korea; 2 Department of Internal Medicine, College of Medicine, Chungbuk National University, Cheongju, Chungbuk, South Korea; Hoffman Heart Institute of the Saint Francis Hospital and Medical Center, UNITED STATES

## Abstract

**Objective:**

Some researchers have studied about early prediction and diagnosis of major adverse cardiovascular events (MACE), but their accuracies were not high. Therefore, this paper proposes a soft voting ensemble classifier (SVE) using machine learning (ML) algorithms.

**Methods:**

We used the Korea Acute Myocardial Infarction Registry dataset and selected 11,189 subjects among 13,104 with the 2-year follow-up. It was subdivided into two groups (ST-segment elevation myocardial infarction (STEMI), non ST-segment elevation myocardial infarction NSTEMI), and then subdivided into training (70%) and test dataset (30%). Third, we selected the ranges of hyper-parameters to find the best prediction model from random forest (RF), extra tree (ET), gradient boosting machine (GBM), and SVE. We generated each ML-based model with the best hyper-parameters, evaluated by 5-fold stratified cross-validation, and then verified by test dataset. Lastly, we compared the performance in the area under the ROC curve (AUC), accuracy, precision, recall, and F-score.

**Results:**

The accuracies for RF, ET, GBM, and SVE were (88.85%, 88.94%, 87.84%, **90.93%**) for complete dataset, (84.81%, 85.00%, 83.70%, **89.07%**) STEMI, (88.81%, 88.05%, 91.23%, **91.38%**) NSTEMI. The AUC values in RF were (98.96%, 98.15%, 98.81%), ET (99.54%, 99.02%, 99.00%), GBM (98.92%, 99.33%, 99.41%), and SVE (**99.61%**, **99.49%**, **99.42%**) for complete dataset, STEMI, and NSTEMI, respectively. Consequently, the accuracy and AUC in SVE outperformed other ML models.

**Conclusions:**

The performance of our SVE was significantly higher than other machine learning models (RF, ET, GBM) and its major prognostic factors were different. This paper will lead to the development of early risk prediction and diagnosis tool of MACE in ACS patients.

## 1. Introduction

From the past few decades, mortality rate of patients with acute coronary syndrome (ACS) has increased [1] and It has become the leading cause of mortality all over the world [2]. According to World Health Organization, acute coronary syndrome is the topmost cause of death worldwide. In Korea, it has become the leading cause of mortality. Acute coronary syndrome is a death causing disease where ST-elevation myocardial Infarction is more fatal than the non-ST-elevation myocardial infarction [3]. Early diagnosis of acute coronary syndrome and prediction of STEMI and NSTEMI traces is very crucial for the patients affected by heart diseases. On the other hand, it is very difficult to accurately predict the solemnity of acute coronary syndrome from the medical dataset as it is dependent on multiple risk factors.

From the Framingham heart study in 1960s [4], the idea for acute coronary syndrome was raised and prediction model for acute coronary syndrome was categorized into two methods namely regression-based methods and machine learning-based methods. There are lots of regressions-based risk prediction models but the most common risk prediction models for early prediction and diagnosis of major adverse cardiovascular events are Thrombolysis In Myocardial Infarction (TIMI) [5] and Global Registry of Acute Coronary Events (GRACE) [6] which are used for risk score prediction of acute coronary syndrome. Both models are using previous medical record for examining and predicting the seriousness of patients, but there are also some drawbacks of these old risk score prediction models as these were designed and implemented around 10 years ago. These models use a few individuals for risk prediction and predict the mortality rate on the basis of these risk predictors. There are also more predictors which can be used to predict the existence of major adverse cardiovascular events (MACE) such as previous medical record and current health status of patient.

There are two methods for diagnosis and prognosis of occurrences of acute coronary syndrome such as clinical methods and design risk prediction model for the diagnosis. Clinical methods for the diagnosis of acute coronary syndrome are Angiography, Electrocardiogram (ECG), Holter monitoring, Echocardiogram, Stress test, Cardiac catheterization, Cardiac computerized tomography (CT) scan, and Cardiac magnetic resonance imaging (MRI) [7]. The other method is to design and develop risk prediction models for early diagnosis and prognosis of ACS using statistical analysis and machine learning algorithms.

Machine learning algorithms improves the prediction accuracy for cardiovascular disease and prevent from unnecessary treatments [8]. Machine learning techniques have overcome the issues of traditional regression-based methods and are popular for diagnosis and prediction of occurrence of MACE. It also overcome the typical data issues and deals with missing values and outliers using data mining techniques. Machine learning techniques relies on non-linier links and interactions between multiple variables and deals with various risk predictors for accurately prediction of risk of patients. This study is also examining the effectiveness of machine learning-based risk prediction techniques to predict the rate of seriousness of patients affected by acute coronary syndrome. Johnson et al. [9] mentioned the importance of machine learning algorithms for prediction and diagnosis of cardiovascular disease. However, the machine learning-based methods have some challenging issues for the prediction of occurrences of MACE for STEMI and NSTEMI groups in patients with acute coronary syndrome as follows. First, there are no specified machine learning or ensemble approaches which gives good results for predictions and dealing with such kind of clinical datasets. In addition, we have to define the specified predictors which are affecting the occurrence of acute coronary syndrome and has a large impact on MACE. Unfortunately, old prediction models are mainly regression-based or their accuracies are ranged between 65 to 84% [10]. Furthermore, these models are dependent on a few risk factors. There are also other risk factors which has more influence on the occurrence of acute coronary syndromes. Furthermore, there are also some other factors which we have to derive from other attributes and have a large impact on acute coronary syndrome.

Therefore, this paper proposes a machine learning-based ensemble classifier with soft voting which can deal with early diagnosis and prognosis of MACE in patients with acute coronary syndrome and provide the best method to deal with the occurrences of cardiac events. The main goal of this paper is to design a risk prediction model for early detection of occurrences of MACE during two-year follow-up after hospital discharge in patients with acute coronary syndrome. Our research contents can also be summarized as follows. First, we use the Korea Acute Myocardial Infarction Registry (KAMIR-NIH) dataset [11] for the experiments and it is separated into two subgroups, STEMI and NSTEMI. Second, we propose a soft voting ensemble classifier using machine learning algorithms such as random forest (RF), extra tree (ET), and gradient boosting machine (GBM), for improving the accuracy of diagnosis and prediction of MACE occurrences [12] such as cardiac death, non-cardiac death, myocardial infarction (MI), re-percutaneous coronary intervention (re-PCI), and coronary artery bypass grafting (CABG). Third, we will specify the risk predictors of MACE for STEMI and NSTEMI groups between previous models and our new model and compare the outcomes of these models. Lastly, we compare the prediction results of occurrences of MACE for STEMI and NSTEMI groups during two-year follow-up after hospital discharge between applied machine learning methods (RF, ET, and GBM) and our soft voting ensemble classifier through the performance measures of accuracy, precision, recall, F1-score, and the area under the ROC curve (AUC).

### 1.1 Related work

Acute coronary syndrome is the fatal disease and it is growing very fast in the whole world. If its diagnosis and early detection will not take place, death ratio will increase very rapidly. So, early detection and risk prediction is mandatory to overcome the death losses from acute coronary syndrome. For this purpose, machine learning algorithms provide the best solutions for diagnosis and early risk predictions of acute coronary syndrome. Jae Kwon Kim et al. [13] used the feature correlation analysis for risk prediction of coronary heart disease by using the neural network but area under the ROC curve was not so high (74.9%) as well as medical experts don’t accept the predictive performance of neural networks because it is trained in a black-box manners. Eka Miranda et al. [14] proposed a model for risk level prediction of acute coronary syndrome using Naïve Bayes Classifier which has good performance (above 80%). They also had mentioned about machine learning based classifiers as the best techniques for prediction of acute coronary syndrome with high validity. Syed Waseem Abbas Sherazi et al. [2] designed a mortality prediction model for patients affected with acute coronary syndrome after their medical discharge and it was averagely improved from Global Registry of Acute Coronary Events (GRACE) risk score model and its results were improved up to 0.08. They had also mentioned that their prognostic factors were different from traditional model. Stephen F. Weng et al. [8] compared the four basic machine learning algorithms such as random forest, gradient boosting machines, logistic regression and neural networks in their experimental work and concluded that machine learning algorithms improved the accuracy of acute coronary syndrome risk prediction and these algorithms could help the patients for preventive treatment. Min-Wei Huang et al. [15] preprocessed the different medical related datasets with categorical, continuous and mixed-type of datasets, and examined that missing value imputation after instance selection can produce better results than imputation alone. But the problem in their work was that they had dealt with missing values, not with data integration, data transformation, and data reduction etc.

Sarab Almuhaideb et al. [16] used the ant colony optimization algorithm to perform classification task in different medical datasets and their predictive accuracy was improved and exceeded 60% in some cases. But this predictive accuracy is not acceptable in medical dataset, as we know that it is very critical data and wrong prediction of model based on medical dataset can lead to the death of patient. Qing Ang et al. [17] preprocessed the medical data by using sigmoid function [18, 19], then self-organizing neural network [20] was used for modeling. Results were compared with clinical diagnosis and concluded that it had almost the same results as clinical diagnosis. H. Benhar et al. [21] thoroughly answered the question that which kind of preprocessing tasks can we do for medical data mining and mentioned that all above mentioned steps of medical data preprocessing are mandatory for efficient output of prediction model. Nongnuch Poolsawad et al. [22] mentioned the top challenging issues of medical data preprocessing and concluded that methods of missing value imputation have no effect on final performance, despite the nature and size of clinical datasets. Uma K et al. [23] followed the sequential steps to preprocess the medical datasets. The preprocessing was carried out on the basis of following steps: i) Data Cleaning, in which they had dealt with data imperfection, missing values, multiple imputation, and noise treatment, ii) Data Integration, iii) Dimensionality Reduction, iv) Discretization. But in their research, they had just mentioned the organized preprocessing cycle to transform the data for machine learning-based risk prediction model, and they did not mention the implementation and results of preprocessing. They had just focused on the theoretical work, not based on the practical and implemented work. Katheleen H. Miao et al. [24] designed and developed an enhanced deep neural network for diagnosis and prognosis of heart disease and their diagnostic results were accurate and reliable, but dataset was not enough to train and validate the results as they used only 303 patient’s dataset.

## 2. Materials and methods

### 2.1 Architecture of our proposed prediction model

**Fig 1:**
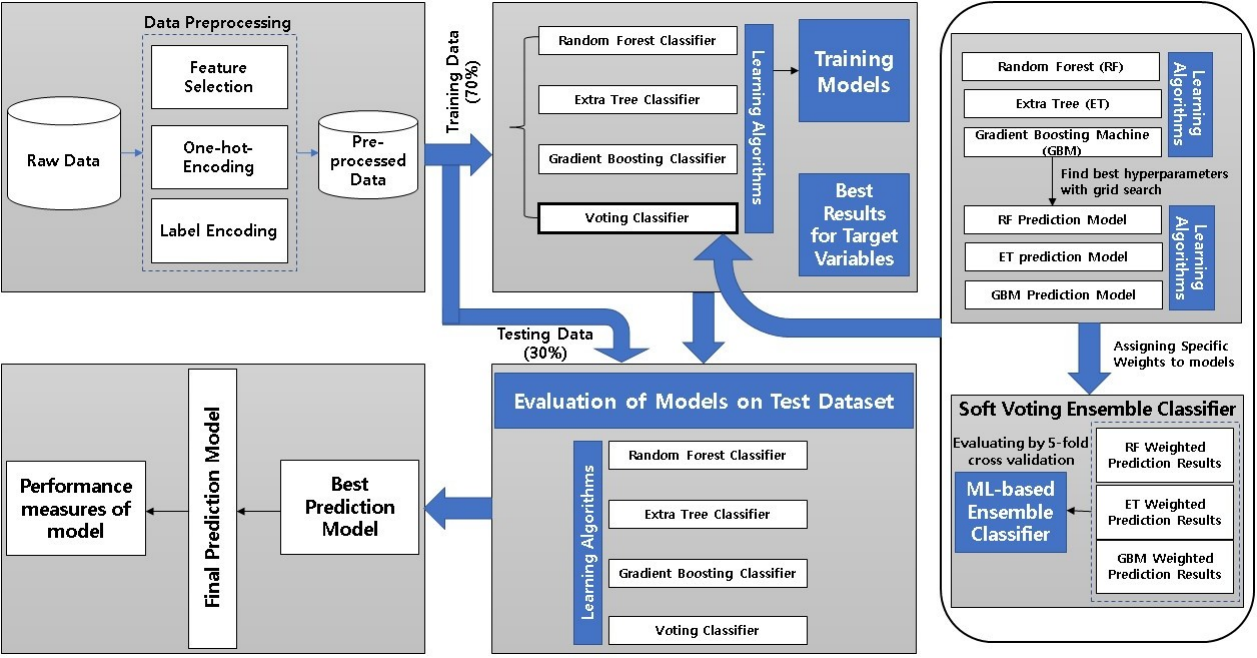
Architecture of our proposed prediction model.

The main steps of our proposed model for early prediction and diagnosis of major adverse cardiovascular events are shown in Fig 1. First step of proposed model is data preprocessing of KAMIR-NIH dataset. In the preprocessing step, we had gone through the feature selection [25] method, in which we dropped the unimportant features from original dataset and use the most important features as our main contributor in this prediction model. We applied one-hot-encoding and label encoding [26] on selected features and prepared our preprocessed dataset for model implementation. Preprocessed data is split into training dataset (70%) and testing dataset (30%) for model training and testing, respectively. The second step of our proposed model is the training of machine learning-based prediction model using the preprocessed dataset. In this step of training model, we had applied three different machine learning models as prediction models e.g. random forest, extra tree, and gradient boosting machine and combined them to design an ensemble model for the best prediction and diagnosis of major adverse cardiovascular events. In our proposed soft voting ensemble classifier, we used the random forest, extra tree, and gradient boosting machine learning algorithms as base classifiers and adjust the hyper parameters by using grid search algorithm to train this model and then was evaluated by 5-fold stratified cross-validation. For the training of this proposed model, we adjusted the weights of these classifiers, because this voting classifier showed the best results on specific weight value. Furthermore, we used the soft voting for our model. We adjusted the tolerance, validation fraction, weight, and other hyper parameters in our proposed model. Hyperparameter tuning is illustrated in Section 4.4. After training of our machine learning-based ensemble model, testing dataset (30%) was applied to verify the performance of our designed model. After the evaluation of model on test data, best hyperparameter values were extracted and finalized the best prediction model by adjusting the hyperparameters. Finally, best prediction model results will be extracted and compared with those of other machine learning models.

#### 2.1.1 Applied machine learning algorithms

For our experimental work we selected three machine learning algorithms named as random forest [27, 28], extra tree [29], and gradient boosting machine [30], and designed our soft voting ensemble classifier based on these three basic models. As compared to other machine learning algorithms, the accuracy of these algorithms was comparatively high, and these were better predicted models for early prediction and diagnosis of acute coronary syndrome.

#### 2.1.2 Proposed ensemble classifier with soft voting

**Fig 2:**
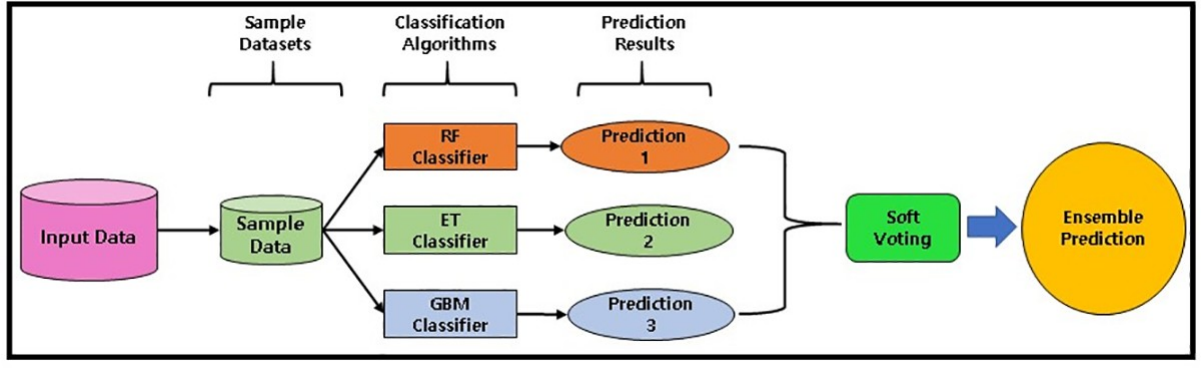
Ensemble classifier with soft voting.

Our designed soft voting ensemble classifier is the combination of multiple classifiers in which decisions are made on the basis of individual decisions which are combined based on probability values to specify that data belongs to a particular class. In the soft voting ensemble method, predictions are weighted on the basis of classifier’s importance and merged them to get the sum of weighted probabilities. The target label with greatest sum of weighted probabilities are selected because it has the greatest voting value (Fig 2). Customized weights can also be used to calculate the weighted average to give more importance and involvement of some specific learning model (base classifier). In contrast of hard voting, soft voting gives better result and performance because it uses the averaging of probabilities [31]. The soft voting ensemble classifier covers up the weakness of individual base classifiers and outperforms the overall results by aggregating the multiple prediction models. The key objective of the ensemble methods is to reduce bias and variance.

### 2.2 Data source

We use the acute coronary syndrome dataset named as Korea Acute Myocardial Infarction Registry (KAMIR-NIH) [11], which is registered in 52 hospitals of Korea and containing all patients’ data from November 2011 to December 2019. We use the two-year dataset in our research work, containing 551 different attributes and 13,104 patients’ medical records with two-year follow-up after hospital discharge. However, there is restriction to share this data because the data is sensitive and not available publicly. Details information about the registry is located at the KAMIR website (http://www.kamir.or.kr). In our data sample, we have all basic medical information of patients such as age, blood pressure, heart rate, height, weight, other diseases record, and previous medical record of patients’ either suffering from any other disease, or have already heart failure, or what is the severity of patient. We have also the complete medication records for heart patients with two- year follow-up. This paper defines major adverse cardiovascular events (MACE) as cardiac death (CD), non-cardiac death (NCD), myocardial infarction (MI), re-percutaneous coronary intervention (re-PCI), and coronary artery bypass grafting (CABG).

### 2.3 Data extraction

**Fig 3:**
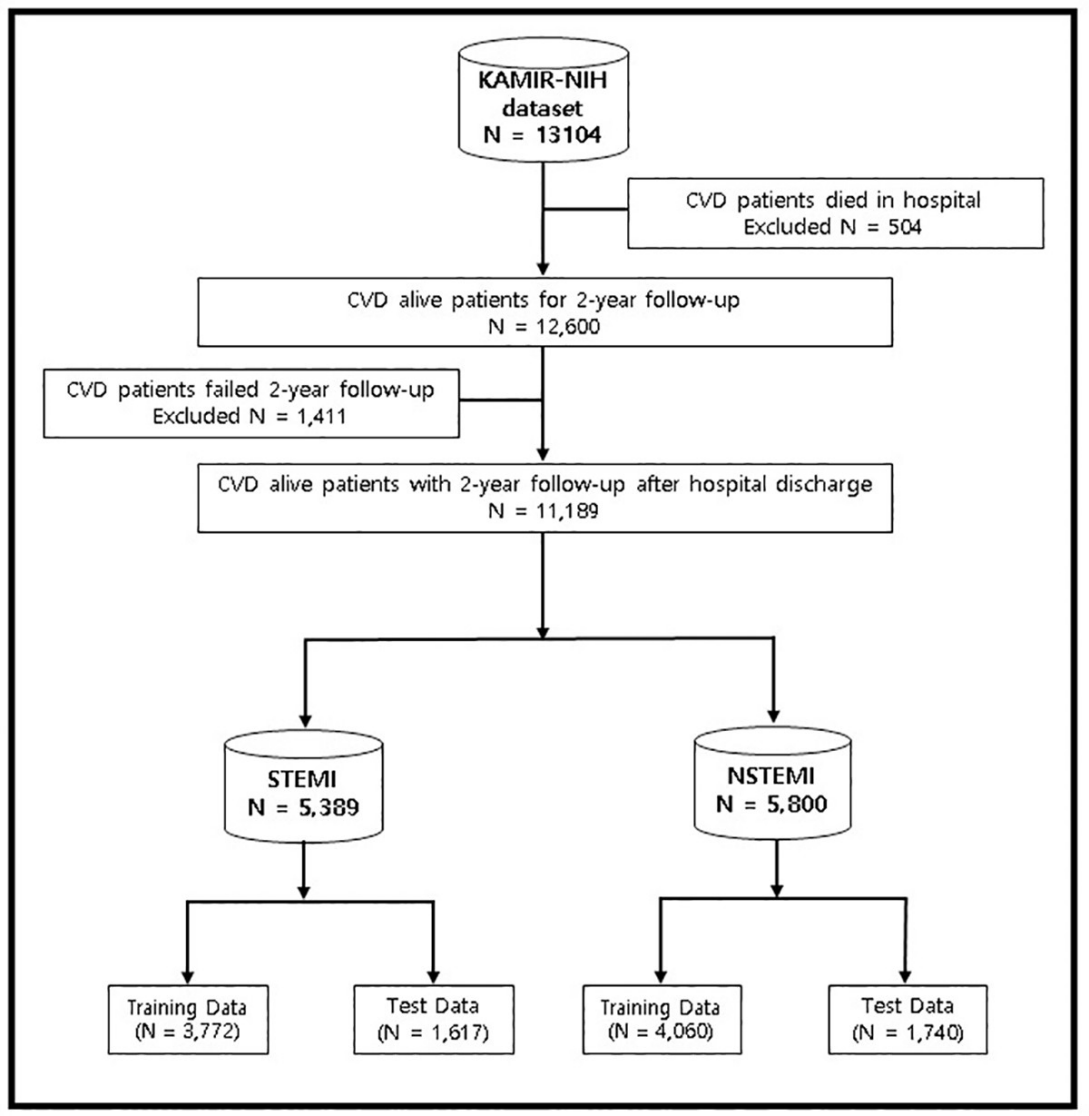
Data extraction from KAMIR-NIH dataset.

Data extraction is the process of extracting or retrieving the data from the unstructured or semi-structure data sources for further data processing to achieve the required results. In case of KAMIR-NIH dataset, it is in raw form and contains inconsistent, noisy, and incomplete data. It also contains data redundancy and outliers. To solve those problems, we preprocess this data into understandable format to get more useful information. We have to apply data extraction methods to extract and manipulate the important features and records from the whole dataset. First of all, we have removed *date* attributes from KAMIR-NIH dataset as these attributes have no impact on the early diagnosis and prognosis of major adverse cardiovascular events. Second, all attributes containing drugs information for patients were eliminated from dataset, because these attributes are not mandatory for the required results and it contains more than 70% null values. Third, all attributes containing more than 70% null values are removed from dataset. After removing all this unnecessary information from the dataset, we extracted the important data from the dataset. Data extraction is illustrated in Fig 3 in which we used KAMIR-NIH dataset (N = 13,104) and excluded all the patients who died in hospital during admission (Excluded N = 504). We also excluded the patients who failed to pursue two-year follow-up (Excluded N = 1,411). After excluding all unnecessary data from the KAMIR-NIH dataset, we had patients with acute coronary syndrome who were alive during two-year follow-up after hospital discharge (N = 11,189). This dataset was then categorized into ST-Elevation Myocardial Infarction (STEMI) (N = 5,389) and Non-ST-Elevation Myocardial Infarction (NSTEMI) datasets (N = 5,800) and then split into training data (70%) and testing data (30%). Complete data extraction processes are illustrated in Fig 3.

### 2.4 Data preprocessing

For preprocessing of KAMIR-NIH dataset, we have classified all attribute features in different categories e.g. categorical features, continuous features, and discrete features. We have defined different preprocessing rules for those different kinds of attributes. For categorical variables, we have applied label encoding [32] as well as one hot encoding [26] to preprocess these variables. For continuous attributes, we have classified the dataset into ranges and then apply label encoding for those defined subclasses. For some categorical and continuous variables containing multiple values, we have applied one hot encoding to easily manage the values of those attributes. One hot encoding is one of the best solutions to manage multiple values and preprocess those attributes containing more than one options. We also have binary valued attributes in our dataset. For these kinds of attributes containing exactly two values, we have converted them into binary form (0 and 1) denoting 0 as No, 1 as Yes.

In our dataset, there were lots of attributes that were not needed in order to apply different algorithms. In order to make our data more specific and error free, we had deleted those attributes from our dataset. For example, some *date* type attributes containing date and time, there is no need to use those attributes in training models. So, we had deleted those attributes. In case of our dataset, some attributes were not present in our dataset, and are very important for prediction models. On the basis of our present dataset, we have derived those attributes by using the other attributes and categorized them to use those attributes. For example, *blood pressure* (*BP*), *body mass index* (*BMI*), *total cholesterol*, and *heart rate* (*HR*) were not present in the dataset, but these were necessary for prediction models. We had derived those attributes from other attributes and categorized them accordingly.

We had also followed the Korean Society of Hypertension guidelines [33, 34] for the categorization of blood pressure and then applied label encoding for data conversion. The *BMI* is calculated as an expression *kg*/*m*^2^ from the patient’ weight (kg) and height (m) and then applied the Korean standards [35] to categorized the BMI values.

We had applied the National Cholesterol Treatment Guidelines [36] to categorize *low*−*density lipoproteins* (*LDL*), *high*−*density lipoproteins* (*HDL*) and *total cholesterol* for Korean patients. The preferred *triglyceride* level is less than 150 mg / dL (1.7 mmol / L), increased borderline is 150–199 mg / dL (1.7–2.2 mmol / L), increased level is 200–499 mg / dL (2.3–5.6 mmol / L), and very high level of triglyceride is 500 mg / dL (5.6 mmol / L) or higher [37]. Without domestic standards, according to the WHO criteria, *hip*−*waist circumference* is an indicator to diagnose abdominal obesity [38] and it indicates the abdominal obesity if WHR>0.9 for males and >0.85 for females.

*C*−*reactive protein* (*high*−*sensitivity CRP*, *hs*−*CRP*) has been used as a predictor of cardiovascular risk in healthy adults [39]. People with high *hs*−*CRP* values have a high risk of acute coronary syndrome and people with low values have low risk. People with higher *hs*−*CRP* results on the upper range of normal have a risk of about 1.5 to 4 times the risk of heart attack than those on the lower side. The Korean Society of Diagnostic Radiology uses the same criteria as the American Heart Association [39, 40] and the US Centers for Disease Control and Prevention. These values are part of the total assessment process for acute coronary syndrome. High levels of blood sugar mean mostly diabetes. However, many diseases and systemic conditions other than diabetes can increase blood sugar. The following information summarizes the meaning of each test result. This summary is based on data from the American Diabetes Association and is classified into normal fasting glucose, pre-diabetes stage, and diabetes [38]. The concentration of serum creatinine is increased by renal dysfunction. The abnormal range for male is >1.2 mg / dl and >1.0 mg / dl for females [41].

In our dataset, we have also dealt with missing values. In medical dataset, it’s very difficult to deal with missing values specially when data is very sensitive. Wrong and inappropriate handling of missing values will lead towards the low prediction of risk factor and vice versa [42]. When we applied machine learning algorithms for risk prediction and early diagnosis and prognosis of acute coronary syndrome, we used different imputation methods for data normalization e.g. mean imputation [43, 44] and k-nearest neighbors (k-NN) imputation [43].

During the data preprocessing, we have examined that some patients have gone through the multiple cardiac events. So, we have categorized the patients undergo the multiple cardiac events into the one cardiac event based on the severity, complications, and effectiveness of that event. For example, a patient has already done CABG and later died because of cardiovascular disease, we have listed that patient into CD, not into CABG.

### 2.5 Hyperparameter tuning

Hyperparameters are parameters for machine learning algorithms whose values are set before training the model, and directly affect the model learning process and efficiency of model. For our experiment, we used four machine learning algorithms such as random forest, extra tree, gradient boosting machine, and our soft voting ensemble classifier. The hyperparameters were tuned for these machine learning-based models before the using of these models, so these models could predict and analyze more accurately and efficiently than other models. Random forest and extra tree were performed more efficiently on the hyperparameters set by default using the *gini criterion* by machine learning library *scikit-learn*. Their accuracies and other performance measures were outperformed on by default hyperparameter tuning rather than tuning by the users. Accuracy of gradient boosting machine was less than 70% on hyperparameters set by default, so it needed to be tuned to get the high results. For GBM, hyperparameters were tuned and then accuracy of GBM was improved up to 90%. Furthermore, hyperparameter tuning was also done for machine learning-based soft voting ensemble model to get the maximum performance. Hyperparameters and their tuning values for each model were illustrated in ***Table 1***.

**Table 1:** Hyperparameter tuning for machine learning algorithms.

Classifier	Hyperparameters and Values
**Random Forest**	Hyperparameters set by default using the gini criterion.
**Extra Tree**	Hyperparameters set by default using the gini criterion.
**Gradient Boosting Machine**	Criterion = ’friedman_mse’ max_depth = 1 min_samples_leaf = 2 min_weight_fraction_leaf = 0.1 presort = ’deprecated’
**Soft Voting Ensemble Classifier**	estimators = [RandomForestClassifier(),ExtraTreesClassifier(),GradientBoostingClassifier(criterion = ’friedman_mse’, max_depth = 1, min_samples_leaf = 2, min_weight_fraction_leaf = 0.1, presort = ’deprecated’)] voting = ’soft’ weights = [9, 6, 2]

### 2.6 Categorizing risk factors

After data extraction and finalizing our dataset for experiment, we can categorize our dataset into different type of attributes named as categorical variables, continuous variables, and discrete variables. For the preprocessing of every type of variable, first we have subdivided our dataset into three categories and then, we have applied different preprocessing methods for each data group so that we can easily apply different algorithms for risk prediction and early diagnose of acute coronary syndrome.

All categorical variables, continuous variables, and discrete variables of KAMIR-NIH dataset are mentioned in ***Table 2*.**

**Table 2:** Variable divisions as categorical and continuous.

Data Type	Variables
**Categorical (25)**	Sex, Chest pain, Dyspnea, Previous chest pain, ECG, Change in ST-segment, Past Medical History, History of Hypertension, History of Diabetes Meletus, History of Dyslipidemia, History of Previous Myocardial Infarction, History of Previous Angina Pectoris, History of Smoking, Family history of Heart Disease, Family History of Early Age Ischemic Heart Disease, PCI, Thrombolysis on admission, Outcome of Thrombolysis, Coronary Angiogram on admission, Coronary Angiogram Result, Initial Diagnosis, CABG, Final Diagnosis, Discharge Type, MACE
**Continuous (28)**	Age, SBP, DBP, Heart Rate, Height, Weight, Abdominal Circumference, BMI, Smoking amount per day, Platelets on admission, LVEF, Glucose on admission, Creatinine on admission, CK-MB, Maximum CK, HDL Cholesterol, LDL Cholesterol, Total Cholesterol, Maximum Troponin I, Maximum Troponin T, Triglyceride, hsCRP, BNP, NT-proBNP, HbA1c, Discharge time SBP, Discharge time DBP, Discharge time Heart Rate
**Discrete (3)**	pre-TIMI, Killip Class, Post-TIMI

*Note: ECG indicates electrocardiography; PCI percutaneous coronary intervention; CABG coronary artery bypass grafting; MACE major adverse cardiovascular events; SBP systolic blood pressure; DBP diastolic blood pressure; BMI body mass index; LVEF left ventricular ejection fraction, CK-MB creatine kinase MB, HDL high-density lipoprotein, LDL low-density lipoproteins, hsCRP high sensitivity C-reactive protein; BNP brain natriuretic peptide / B-type Natriuretic Peptide; NT-proBNP N-terminal pro b-type Natriuretic Peptide; HbA1c hemoglobin A1c; TIMI Thrombolysis In Myocardial Infarction.

### 2.7 Statistical analysis and implementation environments

In statistical analysis, categorical variables are presented as percentage and frequency in dataset, and continuous variables are presented as *mean*±*standard deviation*. Categorical variables are presented as one-hot-encoding method or label encoding method [26], and continuous variables are classified into different ranges and then applied label encoding to transform them into classified values. Scoring of important features is calculated and plotted using feature importance to visualize the important and relevant features for early prediction of occurrence of major adverse cardiovascular events. We also used the unpaired t-test for evaluating the performance significance between STEMI and NSTEMI. Furthermore, we have also considered the important features missing in feature importance and added in our experimental dataset. GRACE and TIMI risk scores were also considered in feature selection.

All statistical analysis and data preprocessing in dataset were applied using SPSS 18 for Windows (SPSS Inc., Chicago, Illinois) [45] and MS Excel for Windows (Microsoft Office 365 ProPlus) [46]. Implementation and development were done in an open source web application of *Jupyter Notebook* in which we can use *scikit-learn* library [47] for machine learning applications and Python Language (Version 3.5) [48].

### 2.8 Performance measures

We applied the dataset to evaluate the accuracy of occurrence of MACE between STEMI and NSTEMI sub-groups of the dataset. We will describe the experimental results of prediction models as a table and the area under the ROC curve (AUC). The performance measures of machine learning-based models will be compared in different matrixes including the area under the ROC curve (AUC), accuracy, precision, recall, F-score and the confusion matrix for actual results versus predicted results.

Confusion matrix mentioned in ***Table 3*** denotes the performance of a classifier in four categories named as *True Positive* (*TP*), *True Negative* (*TN*), *False Positive* (*FP*), and *False Negative* (*FN*) where *True Positive* and *True Negative* are correctly classified, *False Positive* is Type I error and *False Negative* is Type II error.

**Table 3:** Variable divisions as categorical and continuous.

	Predicted Value (Predicted by the test)		
**Actual Value** (Confirmed by experiment)		**Positives**	**Negatives**
	**Positives**	**TP** (True Positive)	**FN** (False Negative)
	**Negatives**	**FP** (False Positive)	**TN** (True Negative)

The formulas for all these performance measures are as follows:

$$Accuracy=\frac{\mathrm{TP}+\mathrm{TN}}{TP+TN+FP+FN}$$ (1)

$$Precision=\frac{\mathrm{TP}}{TP+FP}$$ (2)

$$Recall=\frac{\mathrm{TP}}{TP+FN}$$ (3)

$$F1-score=\frac{2.Precision.Recall}{Precision+Recall}$$ (4)

## 3. Results

In this chapter, we applied the machine learning-based models for early prediction and diagnosis of major adverse cardiovascular events on the basis of ST-elevation myocardial infarction (STEMI) and non-ST-elevation myocardial infarction (NSTEMI) for the patients suffering from acute coronary syndrome. First, we analyzed the baseline characteristics for STEMI and NSTEMI groups on the basis of 24 month’s medical dataset. Then we compared the primary prognostic factors by using the machine learning-based models named as random forest, extra tree, gradient boosting machine, and soft voting ensemble classifier.

### 3.1 Baseline characteristics

After preprocessing the KAMIR-NIH dataset, 11,189 patients’ data was finalized for experimental work including deaths of 300 patients after hospital discharge. First of all, all medical records were subdivided into two groups: ST-elevation myocardial infarction (STEMI) and non-ST-elevation myocardial infarction (NSTEMI) and the baseline characteristics for two groups were summarized in ***Table 4***. In the dataset, there were 172 cardiac deaths, and 128 were non-cardiac deaths. Furthermore, 108 myocardial infarction records were present in dataset in which the number of STEMI and NSTEMI records were 27 and 81, respectively. During two-year follow-up, 292 patients had gone through the re-percutaneous coronary intervention (re-PCI), 13 patients for Coronary Artery Bypass Grafting (CABG), and 110 subjects were re-hospitalized for further medical checkups. Baseline characteristics for both STEMI and NSTEMI subgroups were elaborated in ***Table 4***.

**Table 4:** Baseline characteristics of all subjects (N = 11,189).

Variable	Descriptive Statistics			
	All (N = 11,189)	STEMI (N = 5,389)	NSTEMI (N = 5,800)	p value
Age (years)	62±12.25	61±12.33	64±12.08	<0.001**
Female (%)	24.9 (2783)	20.7 (1115)	28.8 (1668)	0.260
Height (cm)	161±25.83	162±25.59	160±26.0	0.319
Weight (kg)	64±15.17	65±14.97	63±15.30	<0.001**
Abdominal Circumference (cm)	31±42.50	28±41.61	34±43.16	<0.05*
Systolic Blood Pressure (mmHg)	131±29.19	127±30.30	135±27.55	0.391
Diastolic Blood Pressure (mmHg)	79±17.72	77±19.08	81±16.19	0.783
Heart Rate (bpm)	77±18.73	76±19.60	78±17.83	0.874
Pain (typical)	88.2% (9873)	92.6 (4991)	84.2 (4882)	0.136
Dyspnea (yes)	21.8 (2438)	19.2 (1032)	24.2 (1406)	<0.05*
Previous angina before MI symptom (yes)	25.9 (2903)	21.9 (1178)	29.7 (1725)	0.346
Previous myocardial infarction (yes)	7.3 (815)	5.7 (307)	8.8 (508)	<0.001**
Family history of heart disease (yes)	0.7 (83)	0.7 (39)	0.8 (44)	0.248
History of dyslipidemia (yes)	11.8 (1325)	11.2 (604)	12.4 (721)	<0.05*
History of hypertension (yes)	50.1 (5603)	45.6 (2457)	54.2 (3146)	<0.001**
History of diabetes mellitus (yes)	27.2 (3042)	23.7 (1275)	30.5 (1767)	<0.001**
Current smoker (yes)	40.3 (4513)	45.6 (2456)	35.5 (2057)	<0.001**
LV ejection fraction (%)	51.24±13.63	49.49±12.56	52.87±14.36	<0.001**
Glucose (mg/dL)	158±79.44	165±79.55	152±78.80	<0.001**
Creatinine (mg/dL)	1.07±1.12	1.00±0.71	1.14±1.40	<0.001**
Maximum CK (mg/dL)	799.64±1510.51	1145.42±1839.94	478.37±1020.88	<0.001**
Maximum CK-MB (mg/dL)	107.42±157.64	162.62±181.22	56.14±109.40	<0.001**
Maximum Troponon I (mg/dL)	38.96±96.55	61.64±128.57	17.88±41.26	0.264
Maximum Troponon T (mg/dL)	2.35±190.50	0.82±5.25	3.76±264.55	<0.001**
Total Cholestrol (mg/dL)	173±55.87	176±55.02	169±56.46	<0.001**
LDL Cholestrol (mg/dL)	99±51.94	102±51.75	97±51.98	<0.001**
HDL Cholestrol (mg/dL)	40±15.84	40±14.97	40±16.61	<0.05*
Triglyceride (mg/dL)	126±122.88	134±129.33	119±116.16	<0.001**
HsCRP (mg/dL)	0.81±4.58	0.75±3.04	0.86±5.64	<0.001**
BNP (mg/dL)	35.24±255.68	677.17±2563.89	42.75±303.68	<0.001**
NT-proBNP (mg/dL)	1134.09±4398.22	27.15±190.67	1558.63±5553.29	<0.001**
ST segment elevation (yes)	48.6 (5442)	93.6 (5044)	6.9 (398)	<0.05*
ST segment depression (yes)	20.2 (2259)	16.8 (906)	23.3 (1352)	<0.05*
RBBB (yes)	3.5 (393)	2.6 (139)	4.4 (254)	<0.05*
LBBB (yes)	0.9 (105)	0.6 (34)	1.2 (71)	<0.05*
PCI (yes)	91.0 (10187)	97.7 (5265)	84.9 (4922)	0.094

**Note:** N indicates number of patients; MI myocardial infarction; LV left ventricular; CK creatine kinase; CK-MB creatine kinase MB; LDL low-density lipoproteins; HDL high-density lipoproteins; hs-CRP high sensitivity C-reactive protein; BNP brain natriuretic peptide / B-type Natriuretic Peptide; NT-proBNP N-terminal pro b-type Natriuretic Peptide; RBBB right bundle branch block; LBBB left bundle branch block; PCI percutaneous coronary intervention.

**Note:** The single asterisk (*) with p-value denotes that variables are statistically significant as p-value < 0.05 means that there are less than 5% chance of being wrong and double asterisk (**) with p-value denotes that variables are statistically high significant as p-value < 0.001 means that there are less than one in a thousand chance of being wrong in STEMI and NSTEMI groups.

### 3.2 Variable significance / feature importance in early prediction and diagnosis of MACE

**Fig 4:**
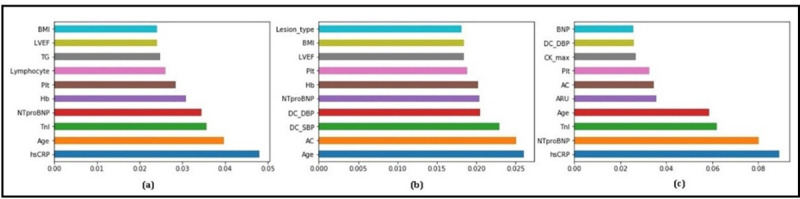
Top 10 primary prognostic factors and their feature importance for. (a) RF; (b) ET; (c) GBM.

The significance of important variables for each model in early prediction and diagnosis of MACE was calculated in percentages. The significance/feature importance for each variable range between 0 and 1: the significance of the most important variable and the least important variable is 1 and 0, respectively. **Fig 4** shows the top 10 most important prognostic factors for each prediction model to predict and diagnose the major adverse cardiovascular events for 24-month follow-up after hospital discharge in patients with acute coronary syndrome. The important risk factors for each prediction model were different and vary from model to model. These primary risk factors for each machine learning-based models were different from traditional regression-based models. Fig 4(A) showed the feature importance for random forest, Fig 4(B) extra tree, and Fig 4(C) gradient boosting machine, respectively. Note that the voting classifier has no feature importance attribute, because this feature importance is available only for tree-based models. The feature importance for the SVE classifier is based on the weighted average of the feature importance of the individual base classifiers.

### 3.3 Comparison of performance measures in prediction models

For the evaluation of risk prediction model of MACE in patients with acute coronary syndrome, we compared the performance of each machine learning-based risk prediction model on the basis of the area under the ROC curve (AUC), accuracy, precision, recall, and F-score. ***Table 5*** showed the performances of applied machine learning models and our designed machine learning-based soft voting ensemble classifier on complete dataset with respect to precision, recall, F-score, and AUC.

#### 3.3.1 Performance measures between MACE and no MACE on complete dataset

Table 5.

**Table 5:** Performance measures for machine learning models on complete dataset (%).

Classifier	Class Labels	Precision	Recall	F-score	AUC
**RF**	**No**	86.395	99.219	92.364	98.486
	**CD**	98.077	98.077	98.077	99.981
	**NCD**	100.00	90.909	95.238	99.880
	**MI**	86.207	58.140	69.444	96.965
	**rePCI**	87.500	88.732	88.112	97.892
	**CABG**	100.00	91.177	95.385	99.987
**ET**	**No**	82.734	97.458	89.494	98.828
	**CD**	100.00	100.00	100.00	100.00
	**NCD**	97.143	91.892	94.444	99.992
	**MI**	89.286	54.348	67.568	99.541
	**rePCI**	91.525	91.525	91.525	98.892
	**CABG**	100.00	97.222	98.592	100.00
**GBM**	**No**	83.648	100.00	91.096	96.824
	**CD**	100.00	100.00	100.00	100.00
	**NCD**	100.00	100.00	100.00	100.00
	**MI**	50.000	9.524	16.000	82.102
	**rePCI**	80.328	92.453	85.965	97.917
	**CABG**	100.00	100.00	100.00	100.00
**SVE**	**No**	88.112	96.923	92.308	98.768
	**CD**	98.182	100.00	99.083	100.00
	**NCD**	97.297	97.297	97.297	99.992
	**MI**	94.118	45.714	61.539	99.387
	**rePCI**	85.507	93.651	89.394	98.866
	**CABG**	100.00	95.238	97.561	100.00

*Note: RF denotes Random Forest; ET Extra Tree; GBM Gradient Boosting Machine; SVE Soft Voting Ensemble; MACE Major Adverse Cardiovascular Events; CD Cadiac Death; NCD Non Cardiac Death; MI Myocardial Infarction, rePCI re-Percutaneous Coronary Intervention; CABG Coronary Artery Bypass Grafting.

#### 3.3.2 Performance measures between MACE and No MACE on STEMI and NSTEMI dataset

***Table 6*** showed the performances of applied machine learning models and our designed machine learning based soft voting ensemble model on STEMI dataset with respect to precision, recall, F-score, and AUC, and ***Table 7*** showed the performance on NSTEMI dataset for all applied models.

**Table 6:** Performance measures for machine learning models on STEMI dataset (%).

Classifier	Class Labels	Precision	Recall	F-score	AUC
**RF**	**No**	90.141	87.671	88.889	95.075
	**CD**	100.00	100.00	100.00	100.00
	**NCD**	100.00	83.333	90.909	100.00
	**MI**	100.00	46.667	63.636	97.279
	**rePCI**	73.171	90.909	81.081	97.686
	**CABG**	78.261	94.737	85.714	99.080
**ET**	**No**	91.667	92.958	92.308	97.586
	**CD**	100.00	83.333	90.909	99.981
	**NCD**	100.00	91.667	95.652	100.00
	**MI**	70.000	50.000	58.333	98.962
	**rePCI**	70.968	84.615	77.193	97.427
	**CABG**	86.957	95.238	90.909	99.899
**GBM**	**No**	98.551	97.143	97.842	99.891
	**CD**	100.00	100.00	100.00	100.00
	**NCD**	75.000	75.000	75.000	99.188
	**MI**	50.000	26.667	34.783	92.290
	**rePCI**	71.429	92.593	80.645	97.476
	**CABG**	100.00	100.00	100.00	100.00
**SVE**	**No**	95.775	95.775	95.775	99.025
	**CD**	100.00	93.333	96.552	100.00
	**NCD**	100.00	90.909	95.238	99.940
	**MI**	100.00	42.857	60.000	100.00
	**rePCI**	76.316	96.667	85.294	99.293
	**CABG**	91.304	100.00	95.455	100.00

*Note: RF denotes Random Forest; ET Extra Tree; GBM Gradient Boosting Machine; SVE Soft Voting Ensemble; MACE Major Adverse Cardiovascular Events; CD Cadiac Death; NCD Non Cardiac Death; MI Myocardial Infarction, rePCI re-Percutaneous Coronary Intervention; CABG Coronary Artery Bypass Grafting.

**Table 7:** Performance measures for machine learning models on NSTEMI dataset (%).

Classifier	Class Labels	Precision	Recall	F-score	AUC
**RF**	**No**	88.608	97.222	92.715	97.550
	**CD**	91.892	100.00	95.775	99.857
	**NCD**	100.00	95.833	97.872	100.00
	**MI**	85.714	40.000	54.546	99.004
	**rePCI**	80.000	90.323	84.849	98.022
	**CABG**	94.444	73.913	82.927	98.530
**ET**	**No**	93.243	92.000	92.617	97.505
	**CD**	100.00	100.00	100.00	100.00
	**NCD**	100.00	96.429	98.182	100.00
	**MI**	63.636	77.778	70.000	95.117
	**rePCI**	93.103	87.097	90.000	98.906
	**CABG**	75.000	81.818	78.261	98.575
**GBM**	**No**	96.000	97.297	96.644	99.892
	**CD**	100.00	100.00	100.00	100.00
	**NCD**	100.00	100.00	100.00	100.00
	**MI**	33.333	28.571	30.769	91.043
	**rePCI**	72.222	74.286	73.239	95.767
	**CABG**	100.00	100.00	100.00	100.00
**SVE**	**No**	93.976	98.734	96.296	99.357
	**CD**	94.286	100.00	97.059	100.00
	**NCD**	100.00	100.00	100.00	100.00
	**MI**	90.909	71.429	80.000	98.070
	**rePCI**	90.909	85.714	88.235	98.589
	**CABG**	94.444	89.474	91.892	99.883

*Note: RF denotes Random Forest; ET Extra Tree; GBM Gradient Boosting Machine; SVE Soft Voting Ensemble; MACE Major Adverse Cardiovascular Events; CD Cadiac Death; NCD Non Cardiac Death; MI Myocardial Infarction, rePCI re-Percutaneous Coronary Intervention; CABG Coronary Artery Bypass Grafting.

As shown in ***Tables 8–10***, the overall accuracy of machine learning-based soft voting ensemble (SVE) classifier is higher (90.93% for complete dataset, 89.07% STEMI, 91.38% NSTEMI) than the other machine learning models such as random forest (88.85%%, 84.81%, 88.81%), extra tree (88.94%, 85.00%, 88.05%), and GBM (87.84%, 83.70%, 91.23%).

**Table 8:** Overall evaluation results for prediction of MACE on complete dataset (%).

Classifier	Accuracy	Precision	Recall	F-measure	AUC
RF	88.85	90.80	90.58	90.17	98.96
ET	88.94	91.31	90.86	90.34	99.54
GBM	87.84	85.27	88.37	84.89	98.92
**SVE**	**90.93**	**92.07**	**91.69**	**90.95**	**99.61**

*Note: RF denotes Random Forest; ET Extra Tree; GBM Gradient Boosting Machine; SVE Soft Voting Ensemble. The boldface values denotes the highest evaluation results among all classifiers.

**Table 9:** Evaluation results for prediction of MACE on STEMI dataset (%).

Classifier	Accuracy	Precision	Recall	F-measure	AUC
RF	84.81	87.54	85.80	85.42	98.15
ET	85.00	87.40	87.04	86.86	99.02
GBM	83.70	88.75	89.51	88.57	99.33
**SVE**	**89.07**	**92.64**	**91.36**	**90.74**	**99.49**

*Note: RF denotes Random Forest; ET Extra Tree; GBM Gradient Boosting Machine; SVE Soft Voting Ensemble. The boldface values denotes the highest evaluation results among all classifiers.

**Table 10:** Evaluation results for prediction of MACE on NSTEMI dataset (%).

Classifier	Accuracy	Precision	Recall	F-measure	AUC
RF	88.81	89.66	89.45	88.63	98.81
ET	88.05	91.97	91.46	91.64	99.00
GBM	91.23	88.94	89.45	89.18	99.41
**SVE**	**91.38**	**93.89**	**93.97**	**93.79**	**99.42**

*Note: RF denotes Random Forest; ET Extra Tree; GBM Gradient Boosting Machine; SVE Soft Voting Ensemble. The boldface values denotes the highest evaluation results among all classifiers.

In fact, ***Tables 8–10*** aggregated the contents of ***Tables 5–7***, respectively. ***Table 8*** presented the overall evaluation of all applied machine learning-based models such as random forest, extra tree, gradient boosting machine, and our proposed soft voting ensemble model for the prediction of major adverse cardiovascular events (CD, NCD, MI, re-PCI, and CABG). ***Tables 9*** and ***10*** presented the evaluation of all applied machine learning based models on STEMI and NSTEMI dataset, respectively.

The accuracies in early prediction models on complete dataset were 88.85%, 88.94%, 87.84%, and 90.93% in RF, ET, GBM, and soft voting ensemble classifier, respectively. The accuracies on STEMI dataset were also 84.81%, 85.00%, 83.70, and 89.07% in RF, ET, GBM, and soft voting ensemble classifier, respectively. Furthermore, the accuracies on NSTEMI dataset were 88.81%, 88.05%, 91.23%, and 91.38% for RF, ET, GBM, and soft voting ensemble model, respectively. Next, the AUC for prediction models were (98.96%, 98.15%, 98.81%) in RF, (99.54%, 99.02%, 99.00%) ET, (98.92%, 99.33%, 99.41%) GBM, and (**99.61%**, **99.49%**, **99.42%**) soft voting ensemble classifier on complete dataset, STEMI, and NSTEMI, respectively. By comparing those models, the accuracy in soft voting ensemble model was averagely improved 2.08%, 4.26%, 2.57% than those in random forest, 1.99%, 4.26%, 3.33% than in extra tree, and 3.09%, 5.37%, 0.15% than in gradient boosting machine on complete dataset, STEMI, and NSTEMI, respectively. The AUC of our machine learning-based soft voting ensemble classifier was also improved from other machine learning models.

The values of all performance measures such as accuracy, precision, recall, F-measure and AUC are illustrated in ***Tables 5–10*** for random forest, extra tree, gradient boosting machine, and our proposed model. These evaluation results showed that our soft voting ensemble classifier outperformed the prediction of MACE from other machine learning models.

### 3.4 Evaluation method

**Fig 5:**
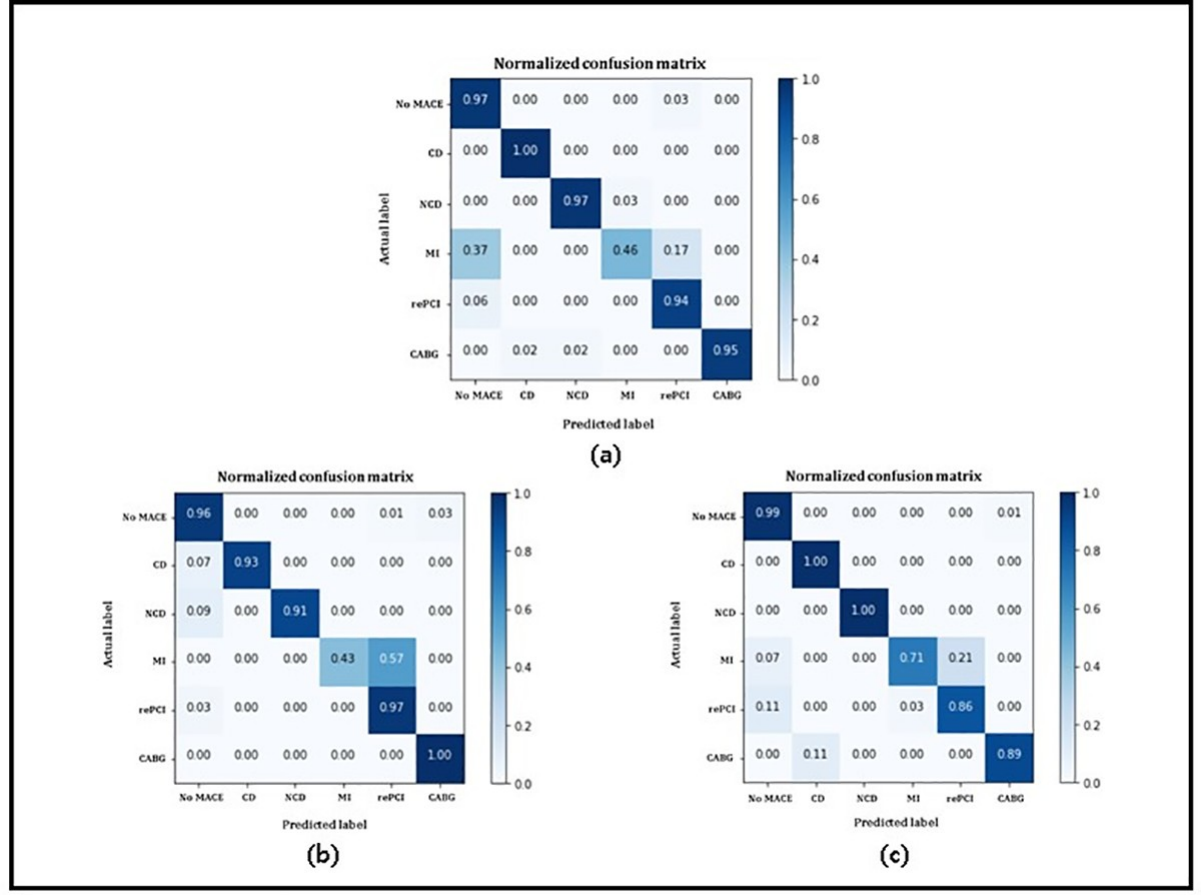
Normalized confusion matrices for proposed soft voting ensemble classifier on. (a) complete dataset; (b) STEMI dataset; (c) NSTEMI dataset.

Confusion matrix is normally used to visualize the performance of algorithms and classifiers. It is a tabular-shaped layout used for visualization of classification results. Normalized confusion matrices for our proposed soft voting ensemble classifier are shown in Fig 5.

In Fig 5, *Actual labe*l on x-axis and *Predicted label* on y-axis represents the actual class labels and predicted class labels, respectively. The diagonal values represent the prediction of all major adverse cardiovascular events. Fig 5A–5C represented the confusion matrix of soft voting ensemble classifier for complete dataset, STEMI, and NSTEMI, respectively. Accuracy value for all MACE outperformed except myocardial infarction (mentioned as 3) because it contained noisy data, outliers, and data redundancy. Other major adverse cardiovascular events were correctly identified, and accuracy was very high which represented that performance of soft voting ensemble was very high. Fig 5 explained the overall performance of our soft voting ensemble model.

We performed the t-test (also known as unpaired t test) for accuracy between STEMI and NSTEMI groups to validate the significance. We analyzed the results from t-test and found that the two-tailed p-value was statistically significant (p-value = 0.0268). The t-value for these groups was 2.9142 (t = 2.9142), the degree of freedom for the test was 6 (df = 6), and standard error of difference was 1.449 (SED = 1.449). ***Table 11*** showed the overall t-test results for each group as follows:

**Table 11:** Unpaired t-test results for accuracy between STEMI and NSTEMI groups.

Group	STEMI	NSTEMI
Mean	85.6450	89.8675
SD	2.3542	1.6897
SEM	1.177	0.8449

*Note: SD denotes Standard Deviation; SEM Standard Error of the Mean.

## 4. Discussion

Machine learning based decision support systems and models for early prediction and diagnosis are becoming widely used in healthcare. These systems help the patients and paramedical staff to improve the decision making and early prediction of MACE occurrences in patients with acute myocardial infarction. Compared with other established algorithms and prediction systems, we found that machine learning algorithms have worked better in prediction and diagnosis of MACE. The best machine learning algorithms were random forest, extra tree, and gradient boosting machine. Other machine learning-based prediction models were also tested but they performed worse and their accuracies were less than these models, so these three models were finalized for our research and applied these models. Unlike other models for risk prediction and early diagnosis, machine learning-based models worked with large set of risk factors and also considered the risk factors used in previous risk prediction models.

In this research article, we applied machine learning algorithms for early prediction and diagnosis of MACE in patients with acute coronary syndrome and used 2-years medical dataset for the experiments. The performance of those models was compared with our machine learning-based soft voting ensemble model. From the experimental results, we found that performance of our soft voting ensemble classifier outperformed those of other machine learning models. Furthermore, prognostic factors for the soft voting ensemble classifier were different from regression-based models. Prognostic factors in our model included the prognostic factors in previous machine learning models as well as newly added prognostic factors (i.e. blood pressure, BMI, and so on). From the experimental results, the prediction results of our soft voting ensemble classifier were significantly higher than other machine learning models on STEMI and NSTEMI groups in patients with acute coronary syndrome in the AUC, precision, recall, F-score, and accuracy (***Tables 5–10***).

The confusion matrix showed that the soft voting ensemble classifier outperformed the results and satisfactory predict all classes except myocardial infarction. The reason of this misclassification was that it contained noisy data and also contained outliers, so our proposed model as well as other machine learning models were unable to accurately predict this cardiac event with high accuracy.

## 5. Conclusion

This paper proposed a soft voting ensemble model for early prediction and diagnosis of MACE occurrences segregation on the basis of ST-elevation myocardial infarction (STEMI) and non-ST-elevation myocardial infarction (NSTEMI) in Korean patients with acute coronary syndrome during 2-year clinical follow up after hospital discharge. Consequently, the performance of our soft voting ensemble classifier for the prediction of MACE occurrences during two-year follow-up in patients with acute coronary syndrome was significantly higher than other machine learning models (RF, ET, GBM) and its major prognostic factors were different. Finally, this machine learning-based ensemble classifier could lead to the development of prediction model of risk score in patients with cardiovascular disease in the future.

### 5.1 Research limitations

There were some limitations in this paper. First, we used Korean medical dataset for prediction and diagnosis hence it is limited for Korean patients and experimental results are more accurate for Korean patients than other races. Second, we used KAMIR-NIH dataset with two-year clinical follow ups of patients, so this model is not perfect for prediction and diagnosis of in-hospital patients and patients with 1,2 or 3 months follow ups.
